# Suitability of CD133 as a Marker for Cancer Stem Cells in Melanoma

**DOI:** 10.31557/APJCP.2021.22.5.1591

**Published:** 2021-05

**Authors:** Philippe Korn, Andreas Kampmann, Simon Spalthoff, Philipp Jehn, Frank Tavassol, Fritjof Lentge, Nils-Claudius Gellrich, Rüdiger Zimmerer

**Affiliations:** 1 *Department of Oral and Maxillofacial Surgery, Hannover Medical School, Hannover, Germany. *; 2 *Department of Oral, Craniomaxillofacial and Facial Plastic Surgery, University Hospital of Leipzig, Germany. *

**Keywords:** Cancer stem cells, CD133, melanoma, xenotransplantation, tumorigenicity

## Abstract

**Objectives:**

CD133 is considered a cancer stem cell (CSC) marker in various malignancies; however, its role as a biomarker of malignant melanoma remains controversial. The present study was conducted to evaluate the suitability of CD133 surface antigen as a CSC marker in melanoma.

**Methods:**

Human melanoma cells were fractionally separated by magnetic cell separation depending on the CD133 phenotype and transplanted into immunodeficient mice to evaluate their tumorigenic capacity. Furthermore, the time until the development of a palpable tumor and the growth rate were measured, and the final tumor volume was assessed after 8 weeks. The immunohistochemical expression of CD133 in the induced neoplasia was then compared using histomorphometry.

**Results:**

Notably, neoplasms were induced in all the groups (n = 48), including in the CD133-negative group. Tumors induced by unsorted cells had the largest volume (p = 0.014) but were detected significantly later in this group (p ≤ 0.001). Interestingly, all explanted tumors expressed CD133, with no significant differences among groups.

**Conclusions:**

In contrast to the results obtained in prior studies, the suitability of CD133 as a CSC marker could not be demonstrated. The current encouraging progress in targeted therapy for malignant melanoma highlights the need to identify more effective targets.

## Introduction

Despite major therapeutic advances and the development of new treatment options, cutaneous melanoma continues to have the highest mortality rate among skin tumors (Cronin et al., 2018; Schvartsman et al., 2019). The encouraging success of targeted therapy and immunotherapy underscores the need to improve our understanding of melanoma tumor biology to facilitate the identification of novel potential targets (Leonardi et al., 2018; Pearlman et al. 2017).

CD133, also known as Prominin-1, is a pentaspan transmembrane glycoprotein encoded by PROM1 located on chromosome 4, which was first described as a marker for hematopoietic progenitor cells (Miraglia et al., 1997; Fargeas et al., 2007). Although the exact physiological function of CD133 remains to be elucidated and its natural ligand is unknown, it appears to be involved in cell differentiation and signal transduction (Sun et al., 2012; Bauer et al., 2011; Irollo and Pirozzi, 2013). Nevertheless, this cell-surface protein is regarded as an important driver of tumor progression and as a putative cancer stem cell (CSC) marker (Glumac and LeBeau, 2018). Our group previously demonstrated the increased ability of CD133-positive (CD133pos) cells to induce neoangiogenesis in vivo (Zimmerer et al., 2016).

In contrast to the stochastic or clonal evolution cancer models, the CSC theory assumes a hierarchical organization of cells, with one subpopulation at the apex, designated as CSCs, capable of tumor initiation and progression; CSCs are characterized by their unlimited self-renewal and lack of growth inhibition, as well as their increased resistance to chemotherapy (Baccelli and Trumpp, 2012).

CD133 has been detected in various types of solid tumors, such as in lung, liver, and colorectal cancers (Glumac and LeBeau 2018). Similarly, melanoma exhibits stemness characteristics through a CD133pos subset. However, the role of CD133 in general and specifically in melanoma remains highly controversial; there is no conclusive evidence regarding whether CD133-negative (CD133neg) subpopulations can initiate tumors (Zimmerer et al., 2016, González-Herrero et al., 2013; Yde et al., 2018; Fomeshi et al., 2016; Rappa et al. 2008; Zimmerer et al., 2013; Roudi et al., 2015; Monzani et al., 2007). In the present study, the relationship between stemness features and CD133 expression was investigated; additionally, the suitability of CD133 as a cancer stem cell marker in malignant melanoma was evaluated. 

## Materials and Methods

*Cell culture*

A metastatic melanoma cell line, D10, characterized by tumor-associated antigen positivity and the HLA-A * 0201 serotype, was used in the present study as previously described by our group (Zimmerer et al. 2013). Briefly, the cells were cultured in Dulbecco’s Modified Eagle Medium supplemented with 10% fetal bovine serum, 100 IU/mL penicillin, and 100 μg/mL streptomycin (all from Biochrom, Berlin, Germany).

*Flow cytometry*

Fluorescence-activated cell sorting (FACS) was performed to identify the CD133pos fraction. Cells in suspension were marked by a specific antibody labelled with allophycocyanin (APC) fluorescent dye (CD133-APC, Miltenyi Biotec GmbH, Bergisch Gladbach, Germany), according to the manufacturer’s protocol. The CD133 phenotype was then determined using the FACSCalibur flow cytometer (BD Biosciences, Franklin Lakes, NJ, USA); evaluation was carried out using CellQuestTM Pro (BD Biosciences) and FlowJoTM v7.6.5 (FlowJo LLC, Ashland, OR, USA) software.

*Cell separation*

The CD133pos and CD133neg phenotypes were separated by Magnetic Activated Cell Sorting (MACS^®^, Miltenyi Biotec GmbH). The cultured D10 cells were first incubated with a CD133-targeted antibody. Next, a secondary antibody with metallic particles (MACS^®^-MicroBeads) against the primary antibody was added. The cells, which were now magnetically labelled, were fractionally separated depending on their CD133 expression profile.

*Animal housing conditions and xenotransplantation*

For in vivo examinations, 24 female NOD/SCID (non-obesity diabetic/severe combined immunodeficiency) mice aged at least 12 weeks and weighing between 19 and 25 g were used. The mice were kept in small groups of up to five animals under a 12-h day/night cycle at a temperature of 22–24°C and constant relative humidity of 60–65%; all animals had unrestricted access to water and feed (1328 hybrid pellet, Altromin Spezialfutter GmbH & Co. KG, Lage, Germany). Three groups of eight animals each were randomized into different xenotransplantation groups according to cell separation status (A: unsorted cells, B: CD133pos, and C: CD133neg); inguinal bilateral subcutaneous injection of 10^5^ tumor cells was carried out under intraperitoneal anesthesia with 75 mg/kg body weight ketamine hydrochloride (Ketamin Gräup, Albrecht GmbH, Aulendorf, Germany) and 25 mg/kg body weight dihydroxylidinothiazine hydrochloride (Rompun^®^, Bayer AG, Leverkusen, Germany). Tumor growth was assessed weekly with a precision caliper according to the longest tumor diameter; after 8 weeks, the test animals were sacrificed by anesthetic overdose and cervical dislocation. The xenografts were subsequently removed, measured, and fixed in 3.5% formaldehyde prior to embedding in paraffin (Merck KGaA, Darmstadt, Germany). The final tumor volume was approximately determined using the following three-axis ellipsoid formula: V =4/3 πabc with a, b, and c representing the radii of the three axes. 

Next, thin sections (5 μm) were prepared and stained with hematoxylin and eosin (both from Merck KGaG) according to established protocols. For immunohistochemical detection of CD133, 5-μm sections were incubated with a rabbit anti-human PROM1/CD133 antibody (Abnova, Taipei, Taiwan), followed by a secondary biotin-conjugated goat anti-rabbit antibody (Dianova, Hamburg, Germany). Next, 3.3′-diaminobenzidine (DAB) (Vector Laboratories, Inc., Burlingame, CA, USA) was added after incubation with streptavidin-conjugated horseradish peroxidase (Dianova), which was followed by counterstaining with hematoxylin for microscopic observation. The primary antibody was omitted from negative controls to ensure no detectable staining.

*Histomorphometry*

To quantify the immunohistochemical staining, the sections were imaged using light microscopy (Leica DM6 with camera DFC7000 T, Leica Mikrosysteme Vertrieb GmbH, Wetzlar, Germany) and assembled into mosaic images covering the entire tumor area using a supported software (Leica Application Suite X (LAS X), Leica Mikrosysteme). Using a previously determined color spectrum, histomorphometric analysis of these images was carried out using the cellSens Dimension 1.14 image analysis software (Olympus, Tokyo, Japan). Immunohistochemically stained regions were subsequently detected using image analysis software and their areas were evaluated in terms of pixels. To account for the different sizes of the individual tumors, the stained areas were considered in relation to the area of counterstained cell nuclei.

*Statistical analysis*

Statistical analyses were performed using SigmaPlot software (Systat Software, Inc., San Jose, CA, USA). Univariate analysis of variance was carried out to detect significant differences between the three comparison groups. The Kruskal–Wallis test was used to compensate for the lack of normal distribution. The Tukey’s test was subsequently used for post-hoc testing to compare individual groups. Variations with p-values <0.05 were considered as statistically significant based on a 95% confidence interval. 

## Results

*Phenotypic characterization*

As a first step, phenotypic characterization of D10 cells according to CD133 epitope expression was performed by flow cytometry; it was determined that 10.7% of these cells were positive for CD133 (CD133pos cells).

*Tumorigenicity*

Three groups of mice were considered for xenotransplantation depending on the phenotype of the injected D10 cells (group A: unsorted cells, group B: CD133pos, and group C: CD133neg). The experimental animals were then periodically examined after the cells were administered into their flanks. Notably, all 48 melanoma cell injections led to tumor manifestations; interestingly, tumor growth was also induced in group C. Thus, there were no differences between the compared groups, indicating that tumorigenicity was not affected by the CD133 phenotype. 

*Tumor growth*

In contrast, the time until a palpable tumor was detected differed. As shown in Figure 2, initial tumor formation was detected significantly later in group A mice (unsorted cells) than in the mice of the other two groups (p ≤ 0.001), whereas, no significant difference was observed between groups B and C (p = 0.992). Although the unsorted cells formed a palpable tumor significantly later, the neoplasms in this group showed the largest volume at the end of the experiment (p = 0.014, Figure 3). Pair-wise differentiation using the post-hoc test showed that this difference was noted only between groups A (unsorted cells) and C (CD133neg) (p = 0.010); other group comparisons did not show significant results (A with B: p = 0.281 and B with C: p = 0.347).

Regarding the course of growth in the host organism during the experiment, no significant difference between the compared groups was detected. Tumor growth (in mm) over time is illustrated in Figure 4. According to analysis of variance, the three compared groups did not significantly differ (p = 0.881).

*Histology and CD133 expression*

Distinct differences between the individual groups could not be detected by light microscopy; there was always a trabecular light-celled growth pattern, accompanied by large core size variance with a conspicuously large number of nucleoli and frequent mitoses. Immunohistochemical staining for CD133 revealed heterogeneous positivity for the examined surface antigen in all tumors (Figure 5); in all groups, tumors showing strong expression of CD133 in addition to those with reduced expression of the marker were detected. However, no specific pattern was observed for this inconsistent distribution. Histomorphometric analysis of the explanted tumors showed no significant difference in CD133 expression (p = 0.07, Figure 6).

**Figure 1:**
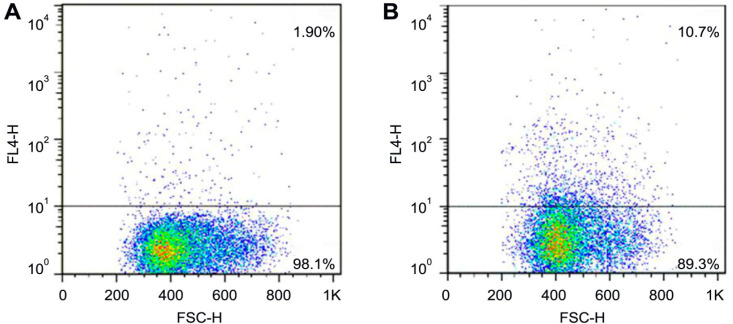
FACS-Analysis of the D10 Cell Line. (A), Autofluorescence of D10 cells; (B), Fluorescence after labelling CD133 (10.7% showed positive signals). FSC-H: forward scatter height; FL4-H: fluorescence emission spectrum 4 height*: chosen cut-off

**Figure 2:**
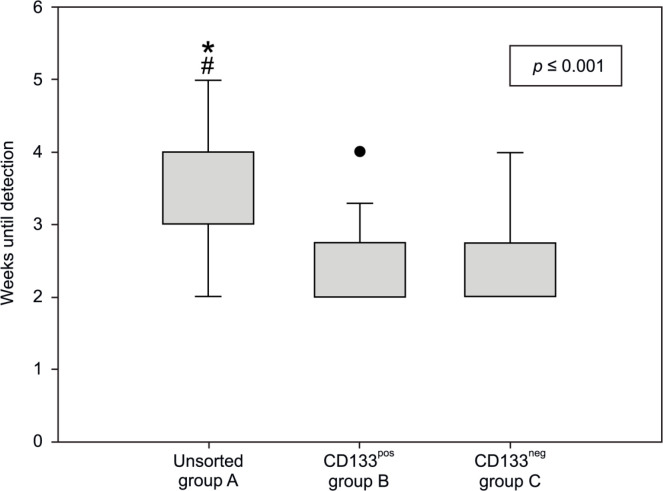
Time until tumor formation in weeks. *, comparison between group A and group B (p = 0.003); #, comparison between group A and group C (p = 0.004)

**Figure 3:**
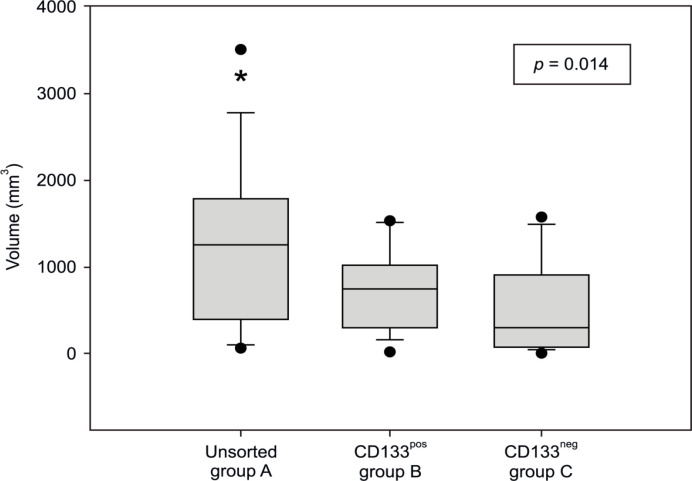
Final Tumor Volume (mm^3^) of the Mice in the Three Groups after Xenotransplantation. All groups showed a significant difference in tumor volume (p = 0.014); * difference between group C and the other groups (post hoc test p = 0.010).

**Figure 4:**
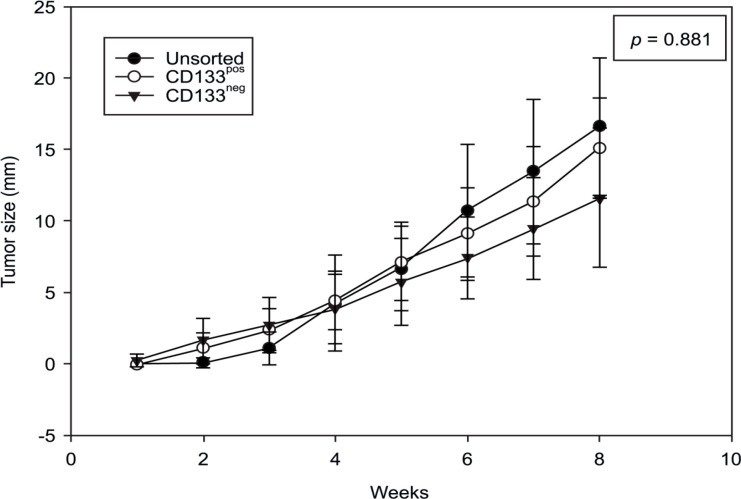
Tumor Size (mm) of the Mice in the Comparison Groups Over Time. All data are represented as the mean values ± standard deviation. There was no significant difference (p = 0.881).

**Figure 5:**
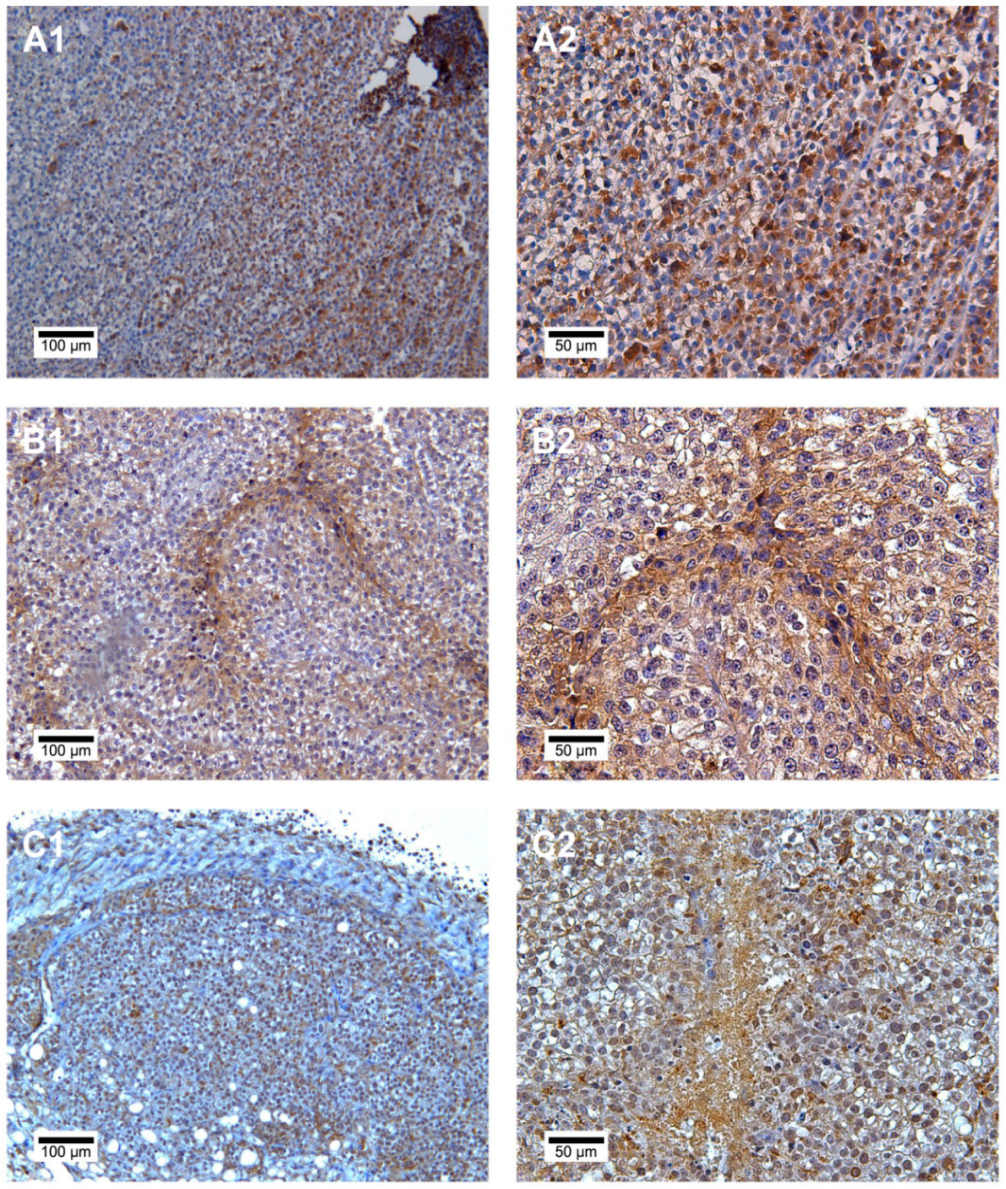
Immunohistochemical Staining of CD133 in Tissues from the Mice of the Three Groups. (A1, B1, C1: 10x magnification); (A2, B2, C2: 20x magnification).Brown: immunohistochemical staining with DAB; blue: counterstaining with hematoxylin

**Figure 6:**
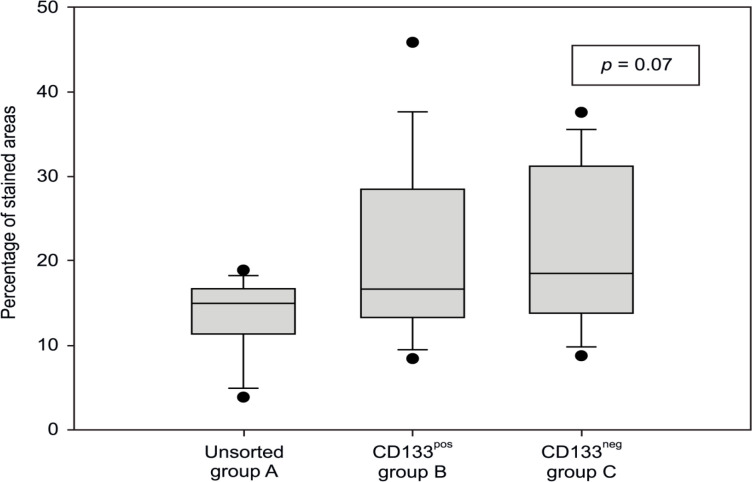
Percentage of CD133-Stained Area in the Three Groups. There was no significant difference between the three groups (p = 0.07).

## Discussion

CD133 is considered the most promising marker for CSCs in melanoma (Madjid et al., 2016). Most studies have reported a rare subset of CD133-positive cells in melanoma comprising less than 1% of total tumor cells (Monzani et al., 2007). The D10 cell line expresses CD133 more frequently than typical melanoma (approximately 10.7%); this high rate appears to be associated with the metastatic state of this cell line (Grasso et al., 2016). However, there are contradictory statements regarding the ability of CD133-negative cells to induce tumors. Monzani (2007) showed that only CD133-positive cells can induce tumors in mice. In our previous study, these results were confirmed using highly immunodeficient NSG mice (Zimmerer et al., 2013). However, other studies found that CD133-negative cells could also induce tumors (Grasso et al., 2016; Quintana et al., 2008); hence, this difference was further investigated using a different murine model. Considering the varying results and numerous factors that can influence this phenomenon, such as host organisms or number of injected cells, CD133 does not appear to be a suitable and specific marker for tumorigenicity.

In addition, the well-known phenomenon of “phenotype switching” causes uncertainty regarding the correct grouping of different cell subsets (Li et al., 2015). Nevertheless, interspecies differences between mice and humans should be considered before evaluating the implications of such animal model-based findings in humans; this is especially because in our study, cutaneous melanoma cells were heterotopically transplanted into the subcutaneous fat of the mouse model. 

Accumulating data have shown that elevated CD133 expression in tumors is associated with more aggressive biological behavior, such as faster growth and poorer survival, as observed in colorectal tumors (Pallini et al., 2011; Horst et al., 2008). Thus, faster tumor growth was anticipated in the CD133-positive group in vivo; however, this was not detectable in our investigation. Moreover, tumors induced by CD133-positive cells were predicted to have a larger tumor volume, which was also not the case in our study. As the group of unsorted cells induced the largest tumor volume, the presence of different cell populations may be conducive for tumor growth. Thus, the presence of CSCs is likely only one factor facilitating tumor growth; the interaction between these cells and neoplastic cells lacking stemness characteristics, non-neoplastic cells within the tumor, as well as the surrounding cells and micromilieu may also drive tumor progression (Somasundaram et al., 2016; Brandner and Haass, 2013).

In summary, all xenotransplanted tumors exhibited detectable CD133 expression via immunohistochemical staining, without significant differences between groups. During tumor growth, this surface characteristic was also expressed in initially CD133neg cells; this may be attributed to the aforementioned “phenotype switching” phenomenon. In general, the plasticity of tumor cells appears to be underestimated. During tumor growth, the influences of intrinsic and extrinsic stress factors repeatedly lead to cellular responses for overcoming suboptimal growth conditions (Leucci et al., 2017); this adaptation may be accompanied by a phenotypic change into a CD133-expressive state. This phenotype instability does not appear to be unidirectional, as once believed, and can pose a major obstacle to the identification of robust CSC markers (Hoek and Goding, 2010). Therefore, combining different markers may be the key to identifying CSCs more reliably and this requires further investigation. There were certain limitations to our study. Monolayer cell cultures can only approximate the complex cellular processes and reaction patterns found in vivo. Even animal models, such as the mouse xenotransplantation model utilized in the present study, harbor uncertainties with regard to the host environment, which differs significantly from the natural environment surrounding human tumor cells. For example, the genomic instability of the xenografts appears to result in chromatic aberrations during growth in the host organism (Ben-David et al., 2017). In addition, the microenvironment in the host organism, particularly in the case of a heterotopic transplant, can affect the expression of surface antigens, thereby influencing the outcome of the study (Grasso et al., 2016; Quintana et al., 2008).

In the present study, CD133neg cells demonstrated tumorigenic potential, in contrast to widely held predictions; conversely, CD133pos cells did not exhibit increased aggressiveness. Thus, in contrast to predictions, CD133 is not an accurate cancer stem cell marker in malignant melanoma. This is consistent with the findings obtained from other studies that critically assess the value of this surface antigen as a specific cancer stem cell marker in malignant melanoma. Further studies using a combination of different markers, while also taking the “phenotype switching” phenomenon into account, should be conducted; this approach may be crucial for reliably detecting cancer stem cells, particularly in malignant melanoma, and for designing true targeted therapies.

## Author Contribution Statement

Philippe Korn: Investigation, Data curation and Writing original draft. Rüdiger Zimmerer and Nils-Claudius Gellrich: Conzeptualisation. Andreas Kampmann, Simon Spalthoff, Philipp Jehn, Frank Tavassol and Fritjof Lentge: Critical Review for important intellectual content. All authors approved the final version of the manuscript. 
